# Non-Melanoma Skin Cancer and Vitamin D: The “Lost Sunlight” Paradox and the Oxidative Stress Explanation

**DOI:** 10.3390/antiox12051107

**Published:** 2023-05-17

**Authors:** Emmanouil Karampinis, Athina-Maria Aloizou, Efterpi Zafiriou, Alexandra Bargiota, Zoi Skaperda, Demetrios Kouretas, Angeliki-Viktoria Roussaki-Schulze

**Affiliations:** 1 Department of Dermatology, University General Hospital Larissa, University of Thessaly, 41500 Larissa, Greece; ekarampinis@uth.gr (E.K.);; 2 Department of Neurology, St. Josef Klinikum Bochum, Ruhr Universität Bochum, 44892 Bochum, Germany; 3 Department of Endocrinology, University General Hospital Larissa, University of Thessaly, 41500 Larissa, Greece; 4 Department of Biochemistry and Biotechnology, University of Thessaly, Viopolis, Mezourlo, 41500 Larissa, Greece

**Keywords:** skin cancer, basal cell carcinoma, cutaneous squamous cell carcinoma, vitamin D, sun exposure, vitamin D deficiency, oxidative stress

## Abstract

UV radiation (UVR) is responsible for inducing both harmful and beneficial effects on skin health. Specifically, it has been reported to disrupt oxidant and antioxidant levels, leading to oxidative stress conditions in skin tissue. This phenomenon might trigger photo-carcinogenesis, resulting in melanoma, NMSC (non-melanoma skin cancer), such as BCC (basal cell carcinoma) and SCC (squamous cell carcinoma), and actinic keratosis. On the other hand, UVR is essential for the production of adequate vitamin D levels, a hormone with important antioxidant, anticancer and immunomodulatory properties. The exact mechanisms implicated in this two-fold action are not well understood, as there still no clear relation established between skin cancer and vitamin D status. Oxidative stress seems to be a neglected aspect of this complex relation, despite its role in both skin cancer development and vitamin D deficiency. Therefore, the aim of the present study is to examine the correlation between vitamin D and oxidative stress in skin cancer patients. A total of 100 subjects (25 with SCC, 26 with BCC, 23 with actinic keratosis, and 27 controls) were assessed in terms of 25-hydroxyvitamin D (25(OH) D) and redox markers such as thiobarbituric acid reactive substances (TBARS), protein carbonyls, total antioxidant capacity (TAC) in plasma, glutathione (GSH) levels and catalase activity in erythrocytes. The majority of our patients revealed low vitamin D levels; 37% of the subjects showed deficiency (<20 ng/mL) and 35% insufficiency (21–29 ng/mL). The mean 25(OH) D level of the NMSC patients (20.87 ng/mL) was also found to be significantly lower (*p* = 0.004) than that of the non-cancer patients (28.14 ng/mL). Furthermore, higher vitamin D levels were also correlated with lower oxidative stress (positive correlation with GSH, catalase activity TAC index and negative correlation with TBARS and CARBS indices). NMSC patients diagnosed with SCC showed lower catalase activity values compared to non-cancer patients (*p* < 0.001), with the lowest values occurring in patients with a chronic cancer diagnosis (*p* < 0.001) and vitamin D deficiency (*p* < 0.001). Higher GSH levels (*p* = 0.001) and lower TBARS levels (*p* = 0.016) were found in the control group compared to the NMSC group, and to patients with actinic keratosis. Higher levels of CARBS were observed in patients with SCC (*p* < 0.001). Non-cancer patients with vitamin D sufficiency showed higher TAC values compared to non-cancer patients with vitamin D deficiency (*p* = 0.023) and to NMSC patients (*p* = 0.036). The above-mentioned results indicate that NMSC patients reveal increased levels of oxidative damage markers compared to control levels, while vitamin D status plays a critical role in the determination of individuals’ oxidative status.

## 1. Introduction

Skin cancer, including melanoma and non-melanoma skin cancer (NMSC) is the most common type of malignancy in the Caucasian population [1]. NMSCs mainly comprise basal cell carcinoma (BCC) and cutaneous squamous cell carcinoma (SCC), as well as rarer skin tumors. BCC arises from the basal layer of the epidermis and its appendages [2], while SCC originates from an uncontrolled proliferation of atypical epidermal keratinocytes [3]. Actinic keratosis results from dysplastic proliferations of keratinocytes with the potential for malignant transformation, and are considered pre-malignant lesions [4]. In sun-damaged skin, BCCs appear clinically and histopathologically in a variety of forms, such as nodular, superficial or pigmented lesions. Patients often complain of an enlarged, nonhealing, bleeding lesion [2]. Bowen’s disease (SCC in situ) usually presents as an erythematous, well-demarcated plaque, while patients with invasive SCC present with a persistent ulcer with hard, raised borders or a wounded skin area [3].

Environmental stimuli, phenotypic characteristics (such as a less protective lighter tone of the skin), and genetic factors contribute to the development of skin cancer, with exposure to ultraviolet radiation (UVR) through sunlight being the most important risk factor for its occurrence [1]. Most risk factors are directly related to sun exposure habits or a person’s sensitivity to solar radiation. UVR, especially UVB (290–320 nm), damages DNA via the formation of cyclobutane pyrimidine dimers (CPDs) and 6–4 photoproducts (6-4PPs). Those products, if not corrected by nucleotide excision repair, interfere with proper base-pairing and obstruct essential cellular processes such as transcription and replication, resulting in progressive gene alterations (including tumor suppressor genes and proto-oncogenes) and ultimately tumor formation [5]. UVR-promoted skin immunosupression and the resulting malfunctioning antigen-presenting mechanisms enhance the photocarcinogenesis process [6]. Moreover, UVR induces an immediate pigment-darkening reaction due to photo-oxidation of preformed melanin, resulting in the appearance of non-cancerous lesions on the skin, such as ephelides, solar lentigines and solar lentigos [7]. However, exposure to sunlight is essential for the skin’s production of vitamin D. Vitamin D is the “sunshine vitamin”, whose metabolites are very important due to their regulatory role in calcium homeostasis and bone metabolic activity, and their protective contribution to the pathogenesis of cancer, heart disease, fractures and autoimmune diseases such as psoriasis [8]. Except for sunlight, nutrition, especially through vitamin-rich fish or mushrooms, is another albeit limited source of vitamin D. A full body UV exposure that causes slight erythema on the skin (1 MED (minimum erythema dose)) is equivalent to an oral intake of vitamin D3 in the range of 250–625 μg (10,000–25,000 IU), while the recommended daily dose of vitamin D is between 400 and 1000 (IU) [9].

The precursor form of vitamin D3, 7-dehydrocholesterol, is present in the plasma membranes of epidermal basal cells, keratinocytes, and dermal fibroblasts, and is converted to pro-vitamin D following exposure to UVB light from the skin. Calciferol, also known as vitamin D, is a broad term for a class of soluble lipids with a four-ring cholesterol skeleton. Whether it comes from food or is produced by the skin, vitamin D is physiologically inactive and needs to be converted through two sequential hydroxylation steps in order to form active metabolites. The main circulating form of vitamin D, 25-hydroxyvitamin D (25 (OH) D), is produced in the liver by the enzyme 25-hydroxylase (via cytochrome P450 2R1 [CYP2R1]). Then, this form is transformed by kidney-mediated 1α-hydroxylase (CYP27B1) to 1,25-dihydroxyvitamin D (1,25(OH)_2_D), the active form of vitamin D [8]. Serum 25(OH)D concentration is determined by the suppression of parathyroid hormone (PTH), the sufficient renal production of 1,25(OH)_2_D, and the intestinal absorption of calcium [10].

The interrelation between vitamin D and skin cancer is controversial [11]. Most studies show an increase in vitamin D levels in skin cancer patients due to intense sun exposure. However, other studies have linked skin cancer to low levels of the hormone, which may be due to the sun protection habits that many skin cancer patients adopt in order to protect their skin from further UV-induced damage [12]. The debate also extends to the therapeutic strategy option of Vitamin D supplementation in patients with BCC. Although meta-analysis has evinced a protective effect of lower vitamin D levels regarding BCC occurrence, there are studies supporting the proposition that higher levels of vitamin D are associated with higher risk of BCC, making the relationship between skin cancer and vitamin D more unclear [13]. A study that focused on the role of vitamin D receptor (VDR) polymorphisms in non-melanoma skin cancer development is also worth mentioning, since it reported a possible relationship between the two [14], highlighting the role of vitamin D in skin cancer.

A possible link between UV exposure, skin carcinogenesis and regulation of vitamin D levels could be the redox status evaluation. Oxidative stress is defined as an imbalance between oxidants and antioxidants in favor of oxidants, a disturbance that can lead to disruption of molecular signaling and/or molecular damage [15]. Reactive oxygen species (ROS) are by-products of aerobic metabolism, including free radicals such as superoxide anion (O_2_^−^) and hydroxyl radical (OH·), as well as hydrogen peroxide (H_2_O_2_), all of which possess intrinsic chemical properties that confer reactivity to different biological targets. ROS are often associated with the concept of oxidative stress, leading to serious damage in lipids, proteins and DNA [16]. To oppose these oxidative conditions, the body is armored with various defense mechanisms, including both enzymatic and non-enzymatic molecules. Among the enzyme biomarkers, superoxide dismutase (SOD), glutathione peroxidase (GPx) and catalase are crucial in maintaining homeostasis for normal cell function. Non-enzymatic mechanisms include proteins that inhibit the formation of new reactive species by binding in metal ions (e.g., iron and copper) or molecules characterized by the ability to rapidly deactivate radicals and oxidants, or repair mechanisms against damage caused by ROS [15,16,17].

UVR exposure is the main initiator of ROS generation in the skin. The action spectrum for ROS production is mainly in the UVA region (320–400 nm), and some spectra overlap with the UVB region. The generation of ROS after UVA and UVB irradiation requires the absorption of photons by endogenous photosensitizer molecules, such as cytochromes, riboflavin, heme and porphyrin [18]. The resulting photosensitizer then reacts with oxygen, leading to the generation of ROS. Additionally, UV light can induce ROS by affecting the enzyme catalase and upregulating the synthesis of nitric oxide synthase (NOS) [19]. After sun exposure to skin cells, damaged biomolecules as well as signal molecules generated by UV exposure can reach the bloodstream and thereby induce systemic oxidative damage [20]. Disruption in redox homeostasis can reduce the ability of the immune system to eliminate cancerous modified cells, as well as disturb antioxidant, apoptotic and DNA-repairing mechanisms that protect against cancer’s initiation, promotion and progression [21,22]. High oxidative stress levels can be detected by certain transient receptor potentials that have been linked to various physiological and pathological processes, and their activation is often accompanied by a significant rise in intracellular Ca^2^+ [23]. Consequently, there is potential to utilize ion channels as novel molecular targets for cancer treatment such as melanoma [24].

Vitamin D is also known for its antioxidant capacities. 1,25(OH)_2_D] binds to VDR, which interacts with the retinoid X receptor (RXR) to form the VDR/RXR heterodimer. Once activated, this unit initiates the sequentially expression of genes interrelated with the antioxidant defense mechanisms, leading to increased expression of both Klotho and Nrf2 proteins, which have powerful antioxidant functions and contribute to the homeostasis of the cell [25]. Consequently, vitamin D opposes the effects emerging from high oxidative damage, while vitamin D deficiency is related to high oxidative stress parameters. In addition to 1,25(OH)_2_D, P450scc (CYP11A1) has the ability to convert vitamin D and lumisterol (a stereoisomer of 7-dehydrocholesterol) into hydroxymetabolites that do not affect calcium levels in the body [26]. Those compounds and their receptors have a crucial role in preventing skin cancer and have been found to be potential targets used for skin cancer treatment [27,28]. These compounds activate protective anti-cancer pathways, such as the Nrf2-antioxidant response and p53-phosphorylation, and induce DNA repair to protect human keratinocytes from DNA damage [29,30]. Additionally, skin cancer patients with low vitamin D levels are found to have a worse prognosis [31]. 

Our retrospective study aims to examine the role of vitamin D and oxidative stress in skin cancer. Oxidative stress parameters associated with skin cancer were examined and correlated with vitamin D levels. This is the first study to assess this interrelation, and may shed light on the heterogenous effects of vitamin D found in skin cancer patients in the literature thus far.

## 2. Materials and Methods

### 2.1. Patient and Control Population

A total of 100 patients who visited the Dermatology Department of Larissa Hospital were enrolled. More specifically, 26 patients with BCC, 25 patients with SCC, 22 patients with actinic keratosis, and 27 control patients with benign lesions, such as lentigines, ephelides and solar lentigos, were included. Besides the skin lesion diagnosis, patients eligible for the study were those who did not have a history of systemic treatment (corticosteroids, immunosuppressive therapy, vitamin D or calcium supplementation, or cholesterol-lowering drugs) or phototherapy application, and with no history of parathyroid or thyroid disorders, autoimmune diseases, anemia, chronic renal or liver disease, or other malignancy. All the patients were adult Caucasians and gave written informed consent. 

Concerning vitamin D status, the study followed the Endocrine Society classification system, which defines, 25-OH D < 20 ng/mL levels as vitamin D deficiency and 25-OH D levels of 21–29 ng/mL as vitamin D inadequacy.

### 2.2. Blood Sample

Blood samples from the patients who agreed to participate in the study were collected via standard venipuncture into blood collection tubes (EDTA, BD Vacutainer^®^, Becton Dickinson, Franklin Lakes, NJ, USA) for the assessment of the oxidative parameters in the erythrocytes and plasma, and into gel tubes for the vitamin D levels evaluation in the form of 25(OH)D. Vitamin D levels were determined with the procedure of chemiluminescence immunoassay (CLIA). The plasma and erythrocyte lysates from the blood samples collected for the determination of the oxidative stress status were immediately placed, after the blood sample fractionation, in liquid nitrogen, and transferred within 3 h to be stored at −80 °C until analysis.

### 2.3. Blood Sample Fractionation and Redox Parameters Determination

The blood samples were centrifuged at 1370× *g* for 10 min at 4 °C. The collected plasma was separated, and the packed erythrocytes were collected and lysed with distilled water (1:1 *v*/*v*), inverted vigorously, and centrifuged at 4000× *g* for 15 min at 4 °C; then, the erythrocyte lysate (RBCL) was collected. 

To measure the concentration of reduced glutathione (GSH), 400 µL of erythrocyte lysate and 400 µL of 5% trichloroacetic acid (TCA) were mixed, inverted vigorously, centrifuged at 15,000× *g* for 5 min at 4 °C, and the supernatant collected. Then, 90 μL 5% TCA was collected, added to the tube and inverted again vigorously and centrifuged as previously described. Finally, the clear supernatant was collected. The determination of the reduced form of GSH was performed using the method of Reddy et al. [32], modified by Veskoukis et al. [33]. According to the proposed method, 20 μL erythrocyte lysate was treated with 5% trichloroacetic acid (TCA), and the mixture was centrifuged in the following conditions: 15,000× *g*, 5 min, 5 °C. The supernatant was collected and 20 μL of it was mixed with 660 μL sodium potassium phosphate buffer (67 mM, pH 8) and 330 μL 5,5′-dithiobis-2 nitrobenzoate (DTNB; 1 mM). The reaction solution was incubated in the dark at room temperature for 15 min, and the optical density was measured at 412 nm.

The catalase activity was assessed using a slightly modified method of Veskoukis et al. [3]; 4 μL RBCL (dilution of 1:10) was mixed with 2991 of 67 mM sodium potassium phosphate buffer (pH 7.4), respectively, followed by incubation at 37 °C for 10 min. Some 5 μL of 30% H_2_O_2_ was added to the mixture, and the rate of the decrease in optical density was measured for 130 s at 240 nm.

For the final calculation of GSH and catalase activity, the hemoglobin concentration needed to be determined. For the hemoglobin concentration of the sample, the hemiglobincyanide (HiCN) method was used. According to the method, we used 5 μL RBCL and 1 mL working hemoglobin reagent (pH of 7.3) composed of potassium hexacyanoferrate (III) (0.607 mmol/L), potassium cyanide (0.767 mmol/L), potassium dihydrogen phosphate (1.03 mmol/L) and 0.05% detergent. The samples were then vortexed and incubated in a dark place for 10 min. Each experiment used 1 mL hemoglobin reagent as a blank. Finally, the optical density was measured at 540 nm. The hemoglobin content was then used to normalize the GSH content and catalase activity of the examined samples.

The collected plasma was used to measure the TAC (total antioxidant capacity), thiobarbituric acid reactive substances (TBARS), and protein carbonyl levels (CARBS). According to the TAC method of Janaszewska and Bartosz [34], a mixture composed of a final volume of 1 mL/20 μL plasma, 480 μL phosphate buffer (10 mM; pH 7.4), respectively, and 500 μL 2,2-diphenyl-1-picrylhydrazyl radical (DPPH, 0.1 mM) solution was centrifuged (15,000× *g*, 3 min) and incubated in the dark at room temperature for 60 min. The optical density of each sample was measured at 520 nm and was presented as mmol of DPPH, reduced to 2,2-diphenyl-1-picrylhydrazine (DPPH:H) by the antioxidants of plasma. 

According to the modified method used for TBARS determination [35], 100 μL plasma, 500 μL 35% TCA and 500 μL Tris–HCl (pH 7.4) were mixed and incubated for 10 min at room temperature. Some 1 mL of Na_2_SO_4_ (2M) and thiobarbituric acid (TBA; 55 mM) was added to the mixture. After 45 min incubation at 95 °C, the samples were transferred at 4 °C for 5 min and vortexed after adding 1 mL of 70% TCA. From each sample, 1 mL was transferred to Eppendorf tubes, and the samples were centrifuged (11,200× *g*, 3 min). Finally, 900 μL of the supernatant was transferred into a plastic cuvette, and the optical density of the MDA (TBA) adduct was determined to be 530 nm.

A slightly modified version of the method of Patsoukis et al. [36] was used for the determination of protein carbonyl content, as previously described by Veskoukis et al. [33] Some 50 µL of plasma and 50 µL of 20% TCA were mixed (each sample had its blank) and then incubated on ice for 15 min. Afterwards, the mixture was centrifuged at 15,000× *g* for 5 min at 4 °C. The supernatant was separated and 0.5 mL of 14 mM DNPH (dissolved in 2.5 N HCL) was added for the samples. Every sample had its own blank that consisted of the same sample volume resuspended with 500 μL 2.5 N HCL without DNPH. Then, the samples and blanks were incubated in the dark at room temperature for 1 h and vortexed every 15 min. After centrifugation (15,000× *g*, 5 min, 4 °C), the supernatant was removed, and the pellets were resuspended with 1 mL TCA (10%). The samples and blanks were again centrifuged (15,000× *g*, 5 min, 4 °C), and the pellets were cleaned with three washes with 1 mL ethanol–ethyl acetate mixture (1:1 *v*/*v*). The pellets were resuspended, while the samples and blanks were centrifuged each time at 15,000× *g* for 5 min at 4 °C. After the third wash, the pellets were resuspended with 1 mL urea (5 M and pH 2.3) and the samples or blanks were vortexed and incubated at 37 °C for 15 min. The samples and blanks were centrifuged (15,000× *g*, 5 min, 4 °C) and their optical density was determined at 375 nm.

### 2.4. Statistical Methods

The data obtained from the experiments were analyzed using the Statistical Package for the Social Sciences, SPSS program. Comparison of numerical variables between the study groups was performed using Student’s *t*-test for comparing independent samples in two groups when normally distributed, and using the Mann–Whitney U-test for independent samples when not normally distributed. Comparison of numerical variables between more than two groups was performed using a one-way analysis of variance test with post hoc multiple two-group comparisons in normal data, and a Kruskal–Wallis test was used for non-normal data. For comparing categorical data, the χ^2^-test was performed. A Shapiro–Wilk test was used to test normality, and Spearman and Pearson correlations were found between the redox markers; 25(OH) D. *p*-values less than 0.05 were considered statistically significant.

## 3. Results

### 3.1. Patients’ Characteristics

The patients’ characteristics, including sex, phototype characteristic according to Fitzpatrick classification, body mass index (BMI), and skin cancer diagnosis, are summarized in Table 1. All patients were asked about their recent or chronic UV exposure time, photoprotective methods they use (suncream application, etc.), and if they had been diagnosed with a UV-induced skin cancerous lesion (such as BCC or SCC) in the past. Recently diagnosed lesions were considered those that had appeared before 1 to 2 months maximum while chronic skin cancer or non-skin cancer patients were considered those that were refused initial therapy or neglected the lesion. Lesions on the cheeks and nose were considered facial lesions, while skin lesions on the scalp and earlobe were the most common non-facial skin lesions.

### 3.2. Vitamin D and Oxidative Stress Parameters

Regarding the assessment of vitamin D levels, 37% of our patients showed deficiency, 35% insufficiency, and 28% sufficiency. The mean redox marker levels and vitamin D levels of the patients are presented in Table 2.

Vitamin D adequacy was considered to be levels >30 ng/mL. Patients that participated in the study lived in Larissa, in the Thessaly region of Greece, located at a latitude of 39.65 and with a mean monthly duration of sunshine of 98.8 h in December and 332.8 h in July [37]. Our patients were examined in the same season in order to account for vitamin D seasonal variation. 

Table 3 shows the results of Spearman and Pearson correlations between the dependent variables of the research. Vitamin D was positively correlated with GSH (*p* < 0.01), catalase activity (*p* < 0.01), and TAC index (*p* < 0.01), while it negatively correlated with TBARS (*p* < 0.01) and CARBS (*p* < 0.01) indices. GSH was also found to be negatively correlated with the TBARS index (*p* < 0.01), while CARBSs negatively correlated with catalase activity (*p* < 0.01).

### 3.3. Vitamin D and Skin Cancer

**Figure 1:**
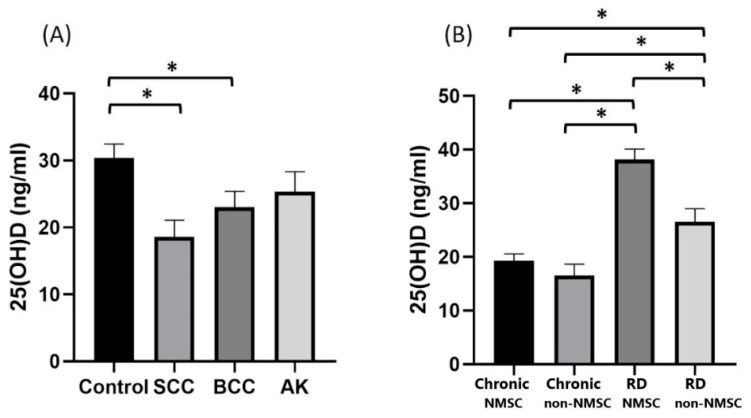
Graphics showing correlation between (**A**) non-melanoma skin cancer subtype and 25(OH) D levels and (**B**) time of skin lesion diagnosis and 25(OH) D levels (SCC: squamous cell carcinoma, BCC: basal cell carcinoma, AK: actinic keratosis, NMSC: non-melanoma skin cancer and RD: recently diagnosed. * p-values of 0.05 or smaller were considered statistically significant using a Kruskal–Wallis test (**A**,**B**).

As shown in Table 4 and Figure 1, vitamin D levels showed statistically significant differences in cancer patients vs. controls (*p* = 0.004), and between cancer types (*p* = 0.006) and time of diagnosis (*p* < 0.001). The mean value of 25(OH) D levels of cancer patients (20.87 ng/mL) was significantly lower than that of non-cancer patients (28.14 ng/mL *p* = 0.004). The median value of 25(OH) D in the control group (28 ng/mL) was significantly higher than that of the subjects with SCC (17.42 ng/mL, *p* = 0.001) and BCC (20.78 ng/mL, *p* = 0.023). The median value of vitamin 25(OH) D of subjects with newly diagnosed non-NMSC skin lesions (39.70 ng/mL) was significantly greater than that of subjects with chronic non-NMSC skin lesions (20.50 ng/mL, *p* < 0.001), recent (28.15 ng/mL, *p* = 0.005) and chronic diagnosis of skin cancer (14.00 ng/mL, *p* < 0.001). Furthermore, the median value vitamin D in the form of 25(OH) D in individuals with a recent skin cancer diagnosis (28.15 ng/mL) was significantly higher than that of individuals with chronic non-NMSC skin lesions (20.50 ng/mL, *p* = 0.046) and chronic skin cancer (14 ng/mL, *p* = 0.002) patients.

### 3.4. Redox Status of the Non-Melanoma Skin Cancer Patients and Controls

**Figure 2:**
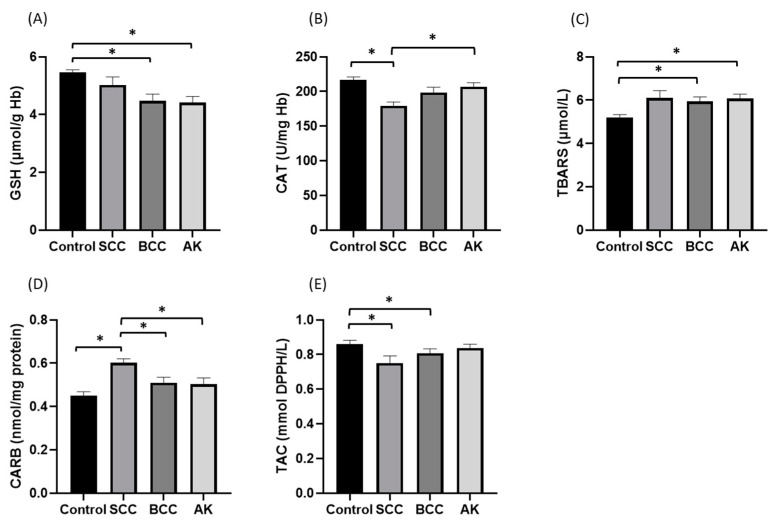
Markers of the redox status in the erythrocytes and plasma of patients with skin cancer as SCC (squamous cell carcinoma) and BCC (basal cell carcinoma), patients with precancerous lesions as actinic keratosis and normal controls. (**A**) erythrocyte-reduced glutathione (GSH) (**B**) erythrocyte catalase (CAT) (**C**) thiobarbituric acid reactive substances (TBARS), (**D**) protein carbonyl (CARB), (**E**) total antioxidant capacity (TAC). * p-values of 0.05 or smaller were considered statistically significant using a one-way ANOVA followed by post hoc multiple two-group comparisons (**A**–**D**) or a Kruskal–Wallis test (**E**).

The GSH marker showed significant associations only with cancer subtype category (*p* = 0.001); the mean GSH value of the control group (5.47 μmol/g Hb) was found to be significantly greater than that of the BCC group (4.48 μmol/g Hb, *p* = 0.002) and that of the actinic keratosis (4.41 μmol/g Hb, *p* < 0.001). Catalase activity levels also showed a significant association with cancer types (*p* < 0.001); the mean catalase activity value of the SCC group (179.32 U/mg Hb) was significantly lower than that of the actinic keratosis group (206.87 U/mg Hb, *p* = 0.007) and the controls (216.54 U/mg Hb, *p* < 0.001) (Figure 2). 

Plasma redox parameters such as TBARS, CARBS and TAC showed statistically significant relationships with the types of cancer (*p* = 0.016, *p* < 0.001 and *p* = 0.036, respectively); the mean TBARS levels in the control group (5.21 μmol/L) were significantly lower than the mean TBARS levels of BCC (5.94 μmol/L, *p* = 0.019) and the actinic keratosis groups (6.07 μmol/L, *p* = 0.005). Concerning the CARBS marker, the mean value of people with SCC (0.60 nmol/mg protein) was significantly higher than that of people with BCC cancer (0.51 nmol/mg protein, *p* = 0.005), actinic keratosis (0.51 nmol/mg protein, *p* = 0.004), and controls (0.45 nmol/mg protein, *p* < 0.001). Finally, in the non-normal distribution of the TAC marker, the control group’s median (0.92 mmol DPPH/L) was significantly higher than that of the SCC (0.83 mmol DPPH /L, *p* = 0.007) and BCC (0.84 mmol DPPH/L, *p* = 0.037) groups (Figure 2).

No statistical significance regarding patients’ sex and oxidative markers such as GSH (*p* = 0.46), catalase activity (*p* = 0.474), TBARS (*p* = 0.336), CARBS (*p* = 0.878) and TAC (*p* = 0.184) was detected.

### 3.5. Oxidative Stress Parameters, Skin Cancer and Vitamin D

**Figure 3:**
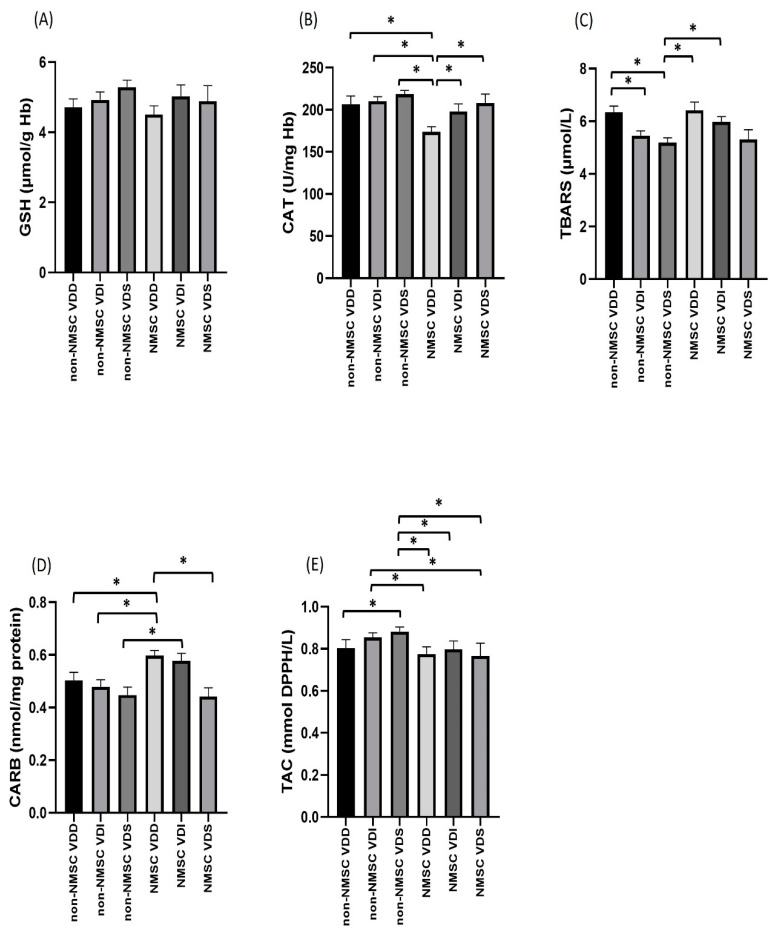
Markers of the redox status in the erythrocytes: (**A**) GSH levels, (**B**) catalase (CAT) activity and plasma (**C**) TBARS, (**D**) CARBS and (**E**) TAC of patients with non melanoma skin cancer (NMSC) and non-NMSC (normal controls with UV-induced lesions and actinic keratosis) and their association with vitamin D levels (VDD: vitamin D deficiency, VDI: vitamin D insufficiency and VDS: vitamin D sufficiency). * p-values of 0.05 or smaller were considered statistically significant using a one-way ANOVA followed by post hoc multiple two-group comparisons (**A**–**D**) or a Kruskal–Wallis test (**E**).

Catalase (*p* < 0.001) and not GSH (*p* = 0.173) showed a statistically significant association with Vitamin D categorization, while the mean value of catalase in vitamin D-deficient cancer patients (173.85 U/mg Hb) was significantly lower than the respective values in cancer patients with insufficiency (196.29 U/mg Hb *p* = 0.019) and vitamin D sufficiency (211.69 U/mg Hb, *p* = 0.001). The catalase marker of cancer patients with deficiency was also found to be lower than the catalase marker of non-cancer patients with insufficiency (210.28 U/mg Hb, *p* < 0.001), deficiency (207.68 U/mg Hb, *p* < 0.001), and non-cancer vitamin D sufficiency (218.72 U/mg Hb, *p* < 0.001). In addition, the mean catalase levels of cancer patients with vitamin D deficiency (196.29 U/mg Hb) were significantly lower (*p* = 0.030) than the mean catalase marker of non-cancer patients with vitamin D deficiency (218.72 U/mg Hb) (Figure 3).

As for the plasma markers of oxidation, TBARS, CARBS and TAC showed a statistically significant association with Vitamin D classification. The mean TBARS levels of non-cancer patients with vitamin D sufficiency (5.19 μmol/L) were significantly lower than those of cancer patients with deficiency (6.42 μmol/L, *p* = 0.021) and vitamin D insufficiency (6.00 μmol/L, *p* = 0.049), as well as non-cancer patients with vitamin D deficiency (6.35 μmol/L, *p* = 0.002). In addition, the mean TBARS index of non-cancer patients with vitamin D insufficiency (5.45 μmol/L) was significantly lower than that of non-cancer patients with vitamin D deficiency (6.35 μmol/L, *p* = 0.032) (Figure 3).

The mean CARBS value in the vitamin D-deficient cancer patients was significantly higher than that of vitamin D-sufficient cancer patients (0.44 nmol/mg protein, *p* = 0.010) but also of non-cancer patients with deficiency (0.48 nmol/mg protein, *p* = 0.046), insufficiency (0.49 nmol/mg protein, *p* = 0.038) and vitamin D sufficiency (0.45 nmol/mg protein, *p* = 0.003). In addition, the mean of the CARBS index of cancer patients with vitamin D insufficiency (0.57 nmol/mg protein) was significantly higher (*p* = 0.043) than the corresponding level of non-cancer patients with vitamin D sufficiency (0.45 nmol/mg protein). The median value of the TAC index of non-cancer patients with vitamin D sufficiency (0.92 mmol DPPH/L) was significantly higher than the corresponding value of cancer patients with deficiency (0.84 mmol DPPH/L, *p* = 0.009), insufficiency (0.84 mmol DPPH/L, *p* = 0.034) and sufficiency (0.79 mmol DPPH/L, *p* = 0.014), as well as the corresponding value of the non-cancer patients with vitamin D deficiency (0.77 mmol DPPH/L, *p* = 0.024). In addition, the median value of the TAC index of non-cancer patients with vitamin D deficiency (0.91 mmol DPPH/L) was significantly higher than the respective value in cancer patients with vitamin D deficiency (0.84 mmol DPPH/L, *p* = 0.034) and cancer patients with vitamin D sufficiency (0.79 mmol DPPH/L, *p* = 0.039) (Figure 3).

### 3.6. Oxidative Stress and Time of the Lesion Appearance

Redox markers of catalase activity, TBARS, CARBS and TAC showed significant association with the time of the lesion appearance. The mean catalase activity of people with a chronic cancer diagnosis (176.47 U/mg Hb) was significantly lower than the corresponding catalase activity of people with a recent cancer diagnosis (206.64 U/mg Hb, *p* = 0.024) and with a recent (215.44 U/mg Hb, *p* < 0.001) and chronic non-cancerous lesions (209.34 U/mg Hb, *p* < 0.001). As for the mean value of the TBARS marker in subjects with a recent diagnosis of a non-cancerous UV-induced lesion (control and actinic keratosis) was significantly lower (5.11 μmol/L) than the corresponding value for individuals with a chronic non-NMSC lesion (control and actinic keratosis) (6.04 μmol/L, *p* = 0.001), and than that of patients with recent (5.79 μmol/L), *p* = 0.035) and chronic cancer diagnosis (6.20 μmol/L, *p* = 0.011).

The mean CARBS value in patients with a chronic diagnosis of cancer (0.59 nmol/mg protein) was significantly higher than the corresponding value of individuals with a recent (0.46 nmol/mg protein, *p* = 0.001) and a chronic sun-related non-NMSC lesion (control and actinic keratosis) (0.49 nmol/mg protein, *p* = 0.003). Finally, the median value of the TAC index of individuals with a recent diagnosis of sun-damage lesion (0.92 mmol DPPH/L) is statistically greater than the corresponding value of the control group with a recent diagnosis of a sun-damage lesion (0.84 mmol DPPH/L, *p* = 0.008), as well as the patient group with chronic NMSC cancer diagnosis (0.84 mmol DPPH/L, *p* = 0.003).

## 4. Discussion

In our study, we retrospectively evaluated the vitamin D levels in the form of 25-OH D of skin cancer patients, patients with precancerous skin lesions such as actinic keratosis, and healthy individuals, and also aimed to investigate the interrelation of vitamin D and redox parameters such as erythrocyte GSH content and catalase activity, plasma TBARS, carbonyls and TAC levels in those patients. The research involved 100 participants, the majority of whom were men with a normal BMI. The sample was almost equally divided between cancer patients and non-cancer patients. The non-cancer patient sample was divided into actinic keratosis and a control group diagnosed with recent or chronic skin lesions related to sun exposure such as lentigines, solar lentigo or ephelides. In half of the cases, the site of damage was on the face, while in most cases no previous cancer lesion was observed. Type II was the most common Fitzpatrick type, meaning that our patients according to the respective classification were prone to easy burns and tan only mildly, due to the smaller amount of melanin that protects the skin against the harmful effects of UVR [38]. Therefore, our findings follow the general rule that the risk of skin cancer or generally sun-related skin damage is higher for people with fair and sun-sensitive skin such as type I or II skin [38], while the Greek population, as a Mediterranean population, mostly consists of III and IV skin types. Concerning UV exposure, it is well established that SCC is mostly associated with cumulative lifetime sun exposure, while intermittent and intense sun exposure is more related to risk of BCC [2,3]. This is the reason for the majority of our SCC patients (20/25) having a history of working in an outdoor occupation, such as farmer or builder, and having chronic skin cancer with neglected cancerous lesions.

The mean vitamin D level of our samples was 24.43 ng/mL, with the majority of them being deficient or insufficient (37% of our patients showed deficiency, 35% insufficiency and 28% sufficiency). These findings are in line with Xyda et al. [39] who, based on demographic information of a large sample of the Greek population, concluded that the prevalence of vitamin D insufficiency (levels < 30 ng/mL) in the Greek sample was 33%, while 40% of Greeks had vitamin D deficiency (levels < 20 ng/mL). According to a systematic review that focused on the vitamin D levels in individuals, residents of sunny countries revealed a high prevalence of low vitamin D status in the Southern European and the Eastern Mediterranean regions, despite the abundant sunshine [40]. Despite the proposed strategies for addressing the deficiency with vitamin enrichment of food and/or with vitamin D supplementation, there is still no clear interpretation of this “lost sunshine” paradox.

The control group of non-cancer patients had higher levels of vitamin D compared to cancer patients. Worth mentioning is the fact that SCC vitamin D-deficient patients with chronic lesions reached very low levels of vitamin D compared to the other categories. Regarding the relationship between skin cancer and vitamin D presented in the literature, the findings are confusing. Mahamat-Saleh et al. [41] in their meta-analysis suggest that high 25 (OH)D levels are associated with increased risk of melanoma and keratocyte origin carcinomas, pointing out that excess UVR exposure is a major confounding factor, as external vitamin D sources do not elevate the risk of skin cancer. Therefore, no clear correlation of vitamin D levels with skin cancer incidence has been shown.

Our results are in line with studies that have found low vitamin D levels in patients with non-melanoma skin cancers. In the study of Tange et al., higher serum 25(OH)D levels were associated with a reduced risk of NMSC in elderly Caucasian men [42]. The results showed that subjects with 25(OH)D > 32 ng/mL appeared to have a 40% lower risk of NMSC, and increasing 25(OH)D levels may be protective against NMSC. Another study in BCC patients from Poland evaluated VDR polymorphisms and showed that vitamin D levels were significantly lower compared to controls, while BCC patients were 35.4% vitamin D-deficient, compared to 10.9% of controls [43]. Some 36% of our BCC patients were also vitamin D-deficient, showing a similar percentage to the aforementioned study. A study in Romanian individuals aiming to characterize sun-related behavior and vitamin D status in 52 BCC patients and 59 controls also revealed lower than expected vitamin D levels [44]. A possible explanation for the low vitamin D levels in this category of patients could be that people with NMSC may exhibit sun avoidance behaviors (suncream application, total body covering with clothes, avoidance of outdoor activities and fear of exposing themselves to sunlight and developing another skin cancer lesion) [44]. Moreover, our study reported that subjects with newly diagnosed skin lesions showed higher levels of vitamin D, while patients with a chronic diagnosis of either cancer or non-cancer skin lesion showed lower levels. This finding is in accordance with the study of Soares et al. [45], which concluded that patients with recently diagnosed NMSC had significantly higher serum levels of 25(OH)D when compared to healthy controls. Nevertheless, the majority of NMSC patients studied had vitamin D insufficiency [45]. This finding could possibly be explained by continuous exposure to sunlight until the discovery of the lesion, and due to the half-life of 25(OH)D being approximately 2–3 weeks.

Concerning the correlation between vitamin D and oxidative stress parameters, we found that participants with higher levels of vitamin D showed higher levels of GSH, catalase activity, and TAC, and lower levels of TBARS and carbonyls. The positive correlation of GSH and Vitamin D was also examined by Jain et al., who showed that GSH status positively upregulates the bioavailability of 25(OH)D via stimulation of vitamin D regulatory genes [46]. Additionally, increased vitamin D levels after vitamin D supplementation were also shown to downregulate TBARS and CARBS levels and upregulate catalase activity [47], showcasing enhanced antioxidant defense mechanisms. Normal serum concentrations of both 25(OH)D and the active form 1,25(OH)_2_D, are crucial for the maintenance of normal cellular functions such as enzymatic reactions, mitochondrial activities, and the proper functioning of the second messenger system [25]. 

In our study, the mean levels of GSH, catalase activity, TBARS, CARBS and TAC were 4.87 μmol/g Hb, 200.39 U/mg Hb, 5.82 μmol/L, 0.52 nmol/mg protein and 0.81 mmol DPPH /L, respectively. In their assessment of redox status in patients with metabolic syndrome and type 2 diabetes, Spanidis et al. [48] used the same methods to calculate their endogenous redox marker levels, and found a similar range and means of those markers as in our study. In both studies, GSH levels varied between patients with different metabolic backgrounds [48], and in patients with different vitamin D profiles. Plasma TAC levels were lower in cancer patients with respect to their matched controls; however, this readout is mostly used to assess the antioxidant capacity of certain foods [49]. TBARS reflect lipid peroxidation, while CARBS is a marker of protein oxidation [16]. As a result, disrupted redox homeostasis can induce TBARS and CARBS production [16], explaining the higher levels of these two redox markers detected herein in cancer patients.

NMSC patients with a diagnosis of SCC presented lower values of catalase activity compared to non-cancer patients, with the lowest values occurring in patients with chronic cancer diagnosis and in cancer patients with vitamin D deficiency, implying higher oxidative damage in this category of patients. Based on the comparison between plasma GSH and catalase in BCC patients and controls, Chaisiriwong et al. [50] found reduced oxidative defenses (reduced catalase and glutathione peroxidase activities in plasma) as well as an increase in a systemic redox marker (urinary 7,8-dihydro-8-oxo-2′-deoxyguanosine) in the cancer group. Similarly, acute exposure of mice skin to UVA and UVB irradiation was also shown to affect markers of oxidative damage and antioxidant defenses in plasma and nonskin tissues such as erythrocytes [20].

Regarding plasma redox markers, lower levels of TBARS appeared in the control group compared to the actinic keratosis group, and also compared to cancer patients diagnosed with BCC. The lowest values were seen in patients (control or actinic keratosis) with recently diagnosed sun damage and with vitamin D sufficiency. As for CARBS, higher levels were observed in patients with SCC cancer. Patients with a chronic diagnosis of cancer showed higher values compared to patients with recent or chronic sun skin damage (control or actinic keratosis). Vitamin D-deficient cancer patients showed higher values of CARBS compared to vitamin D-sufficient cancer patients, and also compared to non-cancer patients. Higher levels of TAC were observed in the control group and in patients with recently diagnosed sun damage compared to cancer patients. The above-mentioned findings are in line with studies that have found increased oxidative damage in the plasma of non-melanoma skin cancer patients [50].

NMSC patients with vitamin D deficiency presented lower catalase activity levels in erythrocytes, and the highest values of CARBS, while control patients with vitamin D sufficiency presented the lowest values of TBARS. The correlation between some of the redox markers measured and vitamin D status has also been investigated for other conditions in the literature. For example, lower levels of vitamin D were associated with oxidative stress in Behcet’s patients, as detected by MDA and NO elevation, as well as decreased GSH, catalase activity and TAC [51]. A similar correlation between vitamin D and oxidative stress conditions was found in our NMSC patients. If there is a dependency between vitamin D deficiency and oxidative stress, it may be interpreted in two ways: (a) if vitamin D deficiency exists first, then due to the deficiency of an antioxidant, oxidative stress occurs; this explanation seems implausible, however, as oxidative stress deficits can be balanced by the many antioxidant mechanisms that control homeostasis and cell function, and (b) if oxidative stress conditions exist first, vitamin D is produced by the skin upon exposure to sunlight in an attempt to balance oxidative stress as an antioxidant, and this vitamin D is then consumed, so the affected individuals present with vitamin D deficiency. The above-mentioned results highly encourage the beneficial impact of vitamin D supplements, antioxidant strategies and their combination in patients with NMSC.

The complex relation between vitamin D and skin cancer is a well-recognized issue with many unanswered questions that necessitates a better approach, possibly by implicating a third parameter, such as oxidative stress. At this point, we need to address the limitations of our study. Firstly, describing the association between vitamin D deficiency and antioxidant status, we did not consider lifestyle/behavior, diet habits, physical activity, and the effect of medical therapy, which are all well-known factors affecting individuals’ redox states. Secondly, concerning cancer and non-cancer patients, we did not assess the disease activity and severity of the lesions. Thirdly, the small sample size represents another factor that might shrink the reliability of our results. Finally, although there were statistically significant differences between patient groups and the control group, the magnitude of these differences was small, limiting the practical implications of those findings.

## 5. Conclusions

The majority of patients studied, in both the NMSC and control groups, appeared with low vitamin D levels (37% presented with deficiency and 35% had insufficiency). This finding is in line with large demographic studies that have assessed vitamin D levels in residents of sunny countries such as Greece, and have defined a paradox between vitamin D deficiency and abundant sunlight. NMSC patients presented with higher systemic oxidative stress markers (lower erythrocyte GSH and catalase activity and higher plasma TBARS and CARBS) than the controls, and their oxidative status was influenced by the subtype of NMSC (BCC or SCC), vitamin D sufficiency, and chronicity of the lesion in question.

## Figures and Tables

**Table 1:** Descriptive characteristics of the study patients.

Variable	Categories-Percentages
Sex	Female (40%, Ν = 40) Male (60%, Ν = 60)
BMI	Underweight (8%, Ν = 8) Normal (55%, Ν = 55) Overweight (30%, Ν = 30) Obese (7%, Ν = 7)
Skin disease category	Skin cancer (51%, Ν = 51) SCC (25%, Ν = 25) BCC (26%, Ν = 26) Precancerous lesion Actinic keratosis (22%, Ν = 22) Control-benign lesions (27%, Ν = 27)
Time of diagnosis	Recently diagnosed NMSC patients (22%, Ν = 22) Chronic NMSC patients (29%, Ν = 29) Recently diagnosed non-NMSC lesion patients (23%, Ν = 23) Chronic non-NMSC lesion patients (26%, Ν = 26)
Site of lesion	Face (56.2%, Ν = 41) Non facial lesions (43.8%, Ν = 32)
Previously diagnosed with skin cancer	No (73.6%, Ν = 53) Yes (26.4%, Ν = 19)
Fitzpatrick Classification	Type 2 (62.6%, Ν = 62) Type 3 (36.4%, Ν = 36) Type 4 (1%, Ν = 1)

**Table 2:** Redox markers and Vitamin D mean values.

Measured Parameter	Mean Value
GSH	4.87 μmol/g Hb (S.D. = 1.13, Range = [2.34, 7.65])
Catalase activity	200.39 U/mg Hb (S.D. = 32.87, Range = [109.83, 282.19])
TBARS	5.82 μmol/L (S.D. = 1.17, Range = [3.05, 9.93])
CARBS	0.52 nmol/mg protein (S.D. = 0.13, Range = [0.21, 0.79])
TAC	0.81 mmol DPPH /L (S.D. = 0.15, Range = [0.14, 0.99])
Vitamin D	24.43 ng/mL (S.D.=12.80, Range= [3, 56])
Vitamin D Classification	Deficiency (37%, Ν = 37) Insufficiency (35%, Ν = 35) Sufficiency (28%, Ν = 28)

GSH: glutathione reduced, TBARS: thiobarbituric acid reactive substances, CARBS: protein carbonyl, TAC: total antioxidant capacity, S.D.: standard deviation.

**Table 3:** Spearman and Pearson correlations between 25(OH) D and oxidative stress parameters.

Variables	GSH	Catalase	TBARS	CARBS	TAC	25(OH) D
GSH	1					
Catalase	0.140	1				
TBARS	−0.273	0.061	1			
CARBS	−0.131	−0.224	0.190	1		
TAC	0.155	−0.032	−0.171	−0.127	1	
25(OH) D	0.277	0.372	−0.429	−0.362	0.222	1

GSH: glutathione reduced, TBARS: thiobarbituric acid reactive substances, CARBS: protein carbonyl, TAC: total antioxidant capacity.

**Table 4:** Vitamin D correlations with skin cancer (presence, type, onset, site).

Variable	Categories	N	Value	Statistics	*p*-Value
Skin disease category	NMSC Control	51 49	Mean value = 20.87 Mean value = 28.14	t (98) = −2.945	0.004
Skin cancer subtype	SCC BCC Actinic Keratosis Control	25 26 22 27	Median = 17.42 Median = 20.78 Median = 22.00 Median = 28.00	H (3) = 12.555	0.006
Time of diagnosis	Recently diagnosed skin cancer patients Chronic skin cancer patients Recently diagnosed non-NMSC lesion patients Chronic non-NMSC lesion patients	22 29 23 26	Median = 28.15 Median = 14.00 Median = 39.70 Median = 20.50	H (3) = 42.257	<0.001
Site of lesion	Facial Non facial	4132	Mean value = 24.00 Mean value = 19.94	t (71) = 1.342	0.184

## Data Availability

The data described in this study are available upon request from the corresponding author.

## References

[1] Apalla Z., Nashan D., Weller R.B., Castellsagué X. (2017). Skin Cancer: Epidemiology, Disease Burden, Pathophysiology, Diagnosis, and Therapeutic Approaches. Dermatol. Ther..

[2] Fania L., Didona D., Morese R., Campana I., Coco V., Di Pietro F.R., Ricci F., Pallotta S., Candi E., Abeni D. (2020). Basal Cell Carcinoma: From Pathophysiology to Novel Therapeutic Approaches. Biomedicines.

[3] Fania L., Didona D., Di Pietro F.R., Verkhovskaia S., Morese R., Paolino G., Donati M., Ricci F., Coco V., Ricci F. (2021). Cutaneous Squamous Cell Carcinoma: From Pathophysiology to Novel Therapeutic Approaches. Biomedicines.

[4] Dodds A., Chia A., Shumack S. (2014). Actinic Keratosis: Rationale and Management. Dermatol. Ther..

[5] Sinha R.P., Häder D.-P. (2002). UV-Induced DNA Damage and Repair: A Review. Photochem. Photobiol. Sci..

[6] Nishisgori C. (2015). Current Concept of Photocarcinogenesis. Photochem. Photobiol. Sci..

[7] Goorochurn R., Viennet C., Granger C., Fanian F., Varin-Blank N., Roy C.L., Humbert P. (2016). Biological Processes in Solar Lentigo: Insights Brought by Experimental Models. Exp. Dermatol..

[8] Heaney R.P. (2008). Vitamin D in Health and Disease. Clin. J. Am. Soc. Nephrol..

[9] Ramasamy I. (2020). Vitamin D Metabolism and Guidelines for Vitamin D Supplementation. Clin. Biochem. Rev..

[10] Dominguez L.J., Farruggia M., Veronese N., Barbagallo M. (2021). Vitamin D Sources, Metabolism, and Deficiency: Available Compounds and Guidelines for Its Treatment. Metabolites.

[11] Morgado-Águila C., Gil-Fernández G., Dávila-Villalobos O.R., Pérez-Rey J., Rey-Sánchez P., Rodríguez-Velasco F.J. (2021). Vitamin D Serum Levels and Non-Melanoma Skin Cancer Risk. PeerJ.

[12] Martin-Gorgojo A., Gilaberte Y., Nagore E. (2021). Vitamin D and Skin Cancer: An Epidemiological, Patient-Centered Update and Review. Nutrients.

[13] Abdelwahab R., Huang R., Potla S., Bhalla S., AlQabandi Y., Nandula S.A., Boddepalli C.S., Gutlapalli S.D., Lavu V.K., Mohammed L. (2022). The Relationship between Vitamin D and Basal Cell Carcinoma: A Systematic Review. Cureus.

[14] Denzer N., Vogt T., Reichrath J. (2011). Vitamin D Receptor (VDR) Polymorphisms and Skin Cancer: A Systematic Review. Dermatoendocrinol.

[15] Sies H. (2020). Oxidative Stress: Concept and Some Practical Aspects. Antioxidants.

[16] Pizzino G., Irrera N., Cucinotta M., Pallio G., Mannino F., Arcoraci V., Squadrito F., Altavilla D., Bitto A. (2017). Oxidative Stress: Harms and Benefits for Human Health. Oxid. Med. Cell. Longev..

[17] Sable K. (2023). The Role of Dietary Antioxidants in Melanoma and Nonmelanoma Skin Cancer. Cutis.

[18] Sharifi-Rad M., Anil Kumar N.V., Zucca P., Varoni E.M., Dini L., Panzarini E., Rajkovic J., Tsouh Fokou P.V., Azzini E., Peluso I. (2020). Lifestyle, Oxidative Stress, and Antioxidants: Back and Forth in the Pathophysiology of Chronic Diseases. Front. Physiol..

[19] Holliman G., Lowe D., Cohen H., Felton S., Raj K. (2017). Ultraviolet Radiation-Induced Production of Nitric Oxide:A Multi-Cell and Multi-Donor Analysis. Sci. Rep..

[20] Rajnochová Svobodová A., Galandáková A., Šianská J., Doležal D., Ulrichová J., Vostálová J. (2011). Acute Exposure to Solar Simulated Ultraviolet Radiation Affects Oxidative Stress-Related Biomarkers in Skin, Liver and Blood of Hairless Mice. Biol. Pharm. Bull..

[21] Obrador E., Liu-Smith F., Dellinger R.W., Salvador R., Meyskens F.L., Estrela J.M. (2019). Oxidative Stress and Antioxidants in the Pathophysiology of Malignant Melanoma. Biol. Chem..

[22] Chen J., Liu Y., Zhao Z., Qiu J. (2021). Oxidative Stress in the Skin: Impact and Related Protection. Int. J. Cosmet. Sci..

[23] Ferrera L., Barbieri R., Picco C., Zuccolini P., Remigante A., Bertelli S., Fumagalli M.R., Zifarelli G., La Porta C.A.M., Gavazzo P. (2021). TRPM2 Oxidation Activates Two Distinct Potassium Channels in Melanoma Cells through Intracellular Calcium Increase. Int. J. Mol. Sci..

[24] Remigante A., Spinelli S., Marino A., Pusch M., Morabito R., Dossena S. (2023). Oxidative Stress and Immune Response in Melanoma: Ion Channels as Targets of Therapy. Int. J. Mol. Sci..

[25] Wimalawansa S.J. (2019). Vitamin D Deficiency: Effects on Oxidative Stress, Epigenetics, Gene Regulation, and Aging. Biology.

[26] Slominski A.T., Brożyna A.A., Zmijewski M.A., Janjetovic Z., Kim T.-K., Slominski R.M., Tuckey R.C., Mason R.S., Jetten A.M., Guroji P. (2020). The Role of Classical and Novel Forms of Vitamin D in the Pathogenesis and Progression of Nonmelanoma Skin Cancers. Adv. Exp. Med. Biol..

[27] Chaiprasongsuk A., Janjetovic Z., Kim T.-K., Schwartz C.J., Tuckey R.C., Tang E.K.Y., Raman C., Panich U., Slominski A.T. (2020). Hydroxylumisterols, Photoproducts of Pre-Vitamin D3, Protect Human Keratinocytes against UVB-Induced Damage. Int. J. Mol. Sci..

[28] Slominski A., Brożyna A., Kim T.-K., Elsayed M., Janjetovic Z., Qayyum S., Slominski R., Oak A., Li C., Podgorska E. (2022). CYP11A1-derived Vitamin D Hydroxyderivatives as Candidates for Therapy of Basal and Squamous Cell Carcinomas. Int. J. Oncol..

[29] Chaiprasongsuk A., Janjetovic Z., Kim T.-K., Jarrett S.G., D’Orazio J.A., Holick M.F., Tang E.K.Y., Tuckey R.C., Panich U., Li W. (2019). Protective Effects of Novel Derivatives of Vitamin D3 and Lumisterol against UVB-Induced Damage in Human Keratinocytes Involve Activation of Nrf2 and P53 Defense Mechanisms. Redox Biol..

[30] Slominski R.M., Raman C., Elmets C., Jetten A.M., Slominski A.T., Tuckey R.C. (2021). The Significance of CYP11A1 Expression in Skin Physiology and Pathology. Mol. Cell. Endocrinol..

[31] Gracia-Darder I., Carrera C., Alamon-Reig F., Puig S., Malvehy J., Podlipnik S. (2022). Vitamin D Deficiency in Melanoma Patients Is Associated with Worse Overall Survival: A Retrospective Cohort Study. Melanoma Res..

[32] Reddy Y.N., Murthy S.V., Krishna D.R., Prabhakar M.C. (2004). Role of Free Radicals and Antioxidants in TB Patients. Indian J. Tuberc.

[33] Veskoukis A.S., Kyparos A., Paschalis V., Nikolaidis M.G. (2016). Spectrophotometric Assays for Measuring Redox Biomarkers in Blood. Biomarkers.

[34] Janaszewska A., Bartosz G. (2002). Assay of Total Antioxidant Capacity: Comparison of Four Methods as Applied to Human Blood Plasma. Scand. J. Clin. Lab. Investig..

[35] Keles M.S., Taysi S., Sen N., Aksoy H., Akçay F. (2001). Effect of Corticosteroid Therapy on Serum and CSF Malondialdehyde and Antioxidant Proteins in Multiple Sclerosis. Can. J. Neurol. Sci..

[36] Patsoukis N., Zervoudakis G., Panagopoulos N.T., Georgiou C.D., Angelatou F., Matsokis N.A. (2004). Thiol Redox State (TRS) and Oxidative Stress in the Mouse Hippocampus after Pentylenetetrazol-Induced Epileptic Seizure. Neurosci. Lett..

[37] Hellenic National Metereological Service Climate Data by City, Sunshine Duration. http://www.Emy.Gr/Emy/El/Agriculture/Agriculture_city?Poli=Larisa.

[38] Merrill S.J., Subramanian M., Godar D.E. (2016). Worldwide Cutaneous Malignant Melanoma Incidences Analyzed by Sex, Age, and Skin Type over Time (1955–2007): Is HPV Infection of Androgenic Hair Follicular Melanocytes a Risk Factor for Developing Melanoma Exclusively in People of European-Ancestry?. Dermatoendocrinol.

[39] Xyda S.E., Kotsa K., Doumas A., Papanastasiou E., Garyfallos A.A., Samoutis G. (2022). Could the Majority of the Greek and Cypriot Population Be Vitamin D Deficient?. Nutrients.

[40] Manios Y., Moschonis G., Lambrinou C.-P., Tsoutsoulopoulou K., Binou P., Karachaliou A., Breidenassel C., Gonzalez-Gross M., Kiely M., Cashman K.D. (2018). A Systematic Review of Vitamin D Status in Southern European Countries. Eur. J. Nutr..

[41] Mahamat-Saleh Y., Aune D., Schlesinger S. (2020). 25-Hydroxyvitamin D Status, Vitamin D Intake, and Skin Cancer Risk: A Systematic Review and Dose–Response Meta-Analysis of Prospective Studies. Sci. Rep..

[42] Tang J.Y., Parimi N., Wu A., John Boscardin W., Shikany J.M., Chren M.-M., Cummings S.R., Epstein E.H., Bauer D.C. (2010). Inverse Association between Serum 25(OH) Vitamin D Levels and Non-Melanoma Skin Cancer in Elderly Men. Cancer Causes Control..

[43] Lesiak A., Norval M., Wodz-Naskiewicz K., Pawliczak R., Rogowski-Tylman M., Sysa-Jedrzejowska A., Sobjanek M., Wlodarkiewicz A., Narbutt J. (2011). An Enhanced Risk of Basal Cell Carcinoma Is Associated with Particular Polymorphisms in the VDR and MTHFR Genes. Exp. Dermatol..

[44] Vornicescu C., Ungureanu L., Șenilă S., Vesa Ș., Cosgarea R., Baican C., Mihu M. (2020). Assessment of Sun-related Behavior and Serum Vitamin&Nbsp;D in Basal Cell Carcinoma: Preliminary Results. Exp. Ther. Med..

[45] Soares A.M., Szejnfeld V.L., Enokihara M.Y., Michalany N., Castro C.H. (2018). High Serum 25-Hydroxyvitamin D Concentration in Patients with a Recent Diagnosis of Non-Melanoma Skin Cancer: A Case-Control Study. Eur. J. Dermatol..

[46] Jain S.K., Parsanathan R., Achari A.E., Kanikarla-Marie P., Bocchini J.A. (2018). Glutathione Stimulates Vitamin D Regulatory and Glucose-Metabolism Genes, Lowers Oxidative Stress and Inflammation, and Increases 25-Hydroxy-Vitamin D Levels in Blood: A Novel Approach to Treat 25-Hydroxyvitamin D Deficiency. Antioxid. Redox Signal..

[47] Paprocki J., Sutkowy P., Piechocki J., Woźniak A. (2021). Association between Vitamin D Supplements, Oxidative Stress Biomarkers, and Hyperbaric Therapy in Patients with Sudden Sensorineural Hearing Loss. Oxid. Med. Cell. Longev..

[48] Spanidis Y., Mpesios A., Stagos D., Goutzourelas n., Baror D., Karapetsa M., Zakynthinos E., Spandidos D.A., Tsatsakis A.M., Leon G. (2016). Assessment of the Redox Status in Patients with Metabolic Syndrome and Type 2 Diabetes Reveals Great Variations. Exp. Ther. Med..

[49] Pompella A., Sies H., Wacker R., Brouns F., Grune T., Biesalski H.K., Frank J. (2014). The Use of Total Antioxidant Capacity as Surrogate Marker for Food Quality and Its Effect on Health Is to Be Discouraged. Nutrition.

[50] Chaisiriwong L., Wanitphakdeedecha R., Sitthinamsuwan P., Sampattavanich S., Chatsiricharoenkul S., Manuskiatti W., Panich U. (2016). A Case-Control Study of Involvement of Oxidative DNA Damage and Alteration of Antioxidant Defense System in Patients with Basal Cell Carcinoma: Modulation by Tumor Removal. Oxid. Med. Cell. Longev..

[51] Omar H.S., Taha F.M., Fouad S., Ibrahim F.A., El Gendy A., Bassyouni I.H., El-Shazly R. (2022). The Association between Vitamin D Levels and Oxidative Stress Markers in Egyptian Behcet’s Disease Patients. Orphanet J. Rare Dis..

